# Spinel of Nickel-Cobalt Oxide with Rod-Like Architecture as Electrocatalyst for Oxygen Evolution Reaction

**DOI:** 10.3390/ma13183918

**Published:** 2020-09-04

**Authors:** Anna Dymerska, Wojciech Kukułka, Marcin Biegun, Ewa Mijowska

**Affiliations:** Department of Nanomaterials Physicochemistry, West Pomeranian University of Technology, Piastow Av. 45, 70-311 Szczecin, Poland; anna.gabryela.dymerska@gmail.com (A.D.); marcin.biegun@zut.edu.pl (M.B.); ewa.borowiak-palen@zut.edu.pl (E.M.)

**Keywords:** spinel, electrocatalyst, oxygen evolution reaction, thermal treatment

## Abstract

The renewable energy technologies require electrocatalysts for reactions, such as the oxygen and/or hydrogen evolution reaction (OER/HER). They are complex electrochemical reactions that take place through the direct transfer of electrons. However, mostly they have high over-potentials and slow kinetics, that is why they require electrocatalysts to lower the over-potential of the reactions and enhance the reaction rate. The commercially used catalysts (e.g., ruthenium nanoparticles—Ru, iridium nanoparticles—Ir, and their oxides: RuO_2_, IrO_2_, platinum—Pt) contain metals that have poor stability, and are not economically worthwhile for widespread application. Here, we propose the spinel structure of nickel-cobalt oxide (NiCo_2_O_4_) fabricated to serve as electrocatalyst for OER. These structures were obtained by a facile two-step method: (1) One-pot solvothermal reaction and subsequently (2) pyrolysis or carbonization, respectively. This material exhibits novel rod-like morphology formed by tiny spheres. The presence of transition metal particles such as Co and Ni due to their conductivity and electron configurations provides a great number of active sites, which brings superior electrochemical performance in oxygen evolution and good stability in long-term tests. Therefore, it is believed that we propose interesting low-cost material that can act as a super stable catalyst in OER.

## 1. Introduction

The fossil fuel combustion products have the most significant impact on air pollution [1], and with the increasing consumption of fossil fuel, it is of great importance for our planet to find some renewable energy resources to replace the traditional ones. These resources should deliver energy without a negative impact on the environment, and good examples could be wind, solar or hydrogen energy. Among green energy, hydrogen and oxygen are easily accessible energy carriers that can be a great answer to the sustained growth of energy requirements for the entire globe [2]. These crucial chemical reagents and fuels are produced via water splitting processes, such as hydrogen and oxygen evolution reactions (HER, and OER, respectively). These reactions are types of electrochemical reactions. Electrocatalysis is involved in electrochemical reactions at the interface of two phases: Electrode and electrolyte [3]. It is essential to mention that under typical operating conditions, only a few materials, used as an electrode, can achieve desirable stability [4]. That is the reason why the anodic, as well as a cathodic reactions, are generally catalyzed. The choice is mostly between noble metal and their oxides such as Pt, Ru, RuO_2_, Ir, IrO_2_. Furthermore, it is worth mentioning that Giordano et al. have recently reported Hf nanoparticles for OER, which exhibiting interesting performance and low over-potential [5].

Nowadays, many studies are focused on finding alternative electrocatalysts, mostly metal-based materials. It is due to their abilities to exhibit great catalytic activity. The main aim is to find an electrode material with better kinetics and stability during electrochemical reactions. Most studies are focused on nanomaterials containing Ru, Ir, and Rh nanoparticles [6], transition metal-based catalysts, e.g., transition-metal oxides (TMOs) [7], hydro(oxy)oxides [8], perovskites [9], spinels [10], metal monoxide structures, for instance, transition-metal dichalcogenides (TMDs) [11], nitrides (TMNs) [12] and phosphides (TMPs) [13], and non-metallic compounds [14]. Among TMOs with chemical formula AB_2_O_4_ and cubic crystallographic structure (especially CoMn_2_O_4_, FeMn_2_O_4_, NiCo_2_O_4_, NiMn_2_O_4_ and ZnCo_2_O_4_) have been claimed as promising anode materials for electrochemical use, e.g., LIBs, supercapacitors, and electrocatalysts mainly in OER [15]. These chemical compounds belong to the spinel group [16]. 

The name, “spinel”, is used for all minerals with the cubic structure and general composition AB_2_X_4_, where A and B are metal cations (at the +1/+2/+4, and +2/+3 valences, respectively) and X is chalcogens anion (at the −1/−2/−3 valence, mostly oxygen). It is worth mentioning that the cations A and B can also be the same element, but with different valences, e.g., magnetite—Fe_3_O_4_ (with one atom of iron at 2+ and two atoms at +3 valences state) [17]. So far, 95 notable spinels have been reported [17]. Noteworthy, they have shown better properties in comparison with their simple counterpart—single metal TMOs. The explanation is the synergistic and complementary effect [18]. Their most popular applications are energy storage [19] (because of their variable chemical valances and redox capacitance), data storage [20] (they have demonstrated outstanding magnetism properties), optical devices like lasers [21] (due to electrochemical luminescence and photoluminescence), various reaction catalysis, e.g., HER [22], OER [23], oxygen reduction reaction (ORR) [24] (they have controllable composition, valence, structure, and morphology).

NiCo_2_O_4_ is an inverse spinel, which means the divalent cations swap place with half of the trivalent ones in its structure. In that case, the cations M^2+^ occupy octahedral sites [25]. Nickel-cobalt oxide has the $Fd3\bar{m}$ space group. In this compound, nickel is located on the octahedral sites (16d) and cobalt is in the octahedral and tetrahedral sites (16d, and 8a, respectively). It is claimed that the pairs of their redox cations (Ni^3+^/Ni^2+^, Co^3+^/Co^2+^) are distributed in the oxidation state of this spinel structure [26]. It has many advantages, such as good electrochemical performance (such as significant specific capacitance, high activity, splendid stability) and is noteworthy that it is environmentally friendly [27]. It is crucial to know that electrochemical properties strongly depend on its structure/shape. There are many reported methods of syntheses of NiCo_2_O_4_ particles that have found application in electrochemistry, for example, mesoporous nanoneedles with different shapes for electrochemical capacitor electrodes, that show outstanding capacitance of 932 Fg^−1^ (at 2.0 Ag^−1^) and an imperceptibly small drop of specific capacitance after 3000 cycles (at 2.0 Ag^−1^) [28]. The mesoporous flake-like nanoparticles NiCo_2_O_4_ for supercapacitor electrodes, with good electrochemical properties: 1125 Fg^−1^ (at 0.05 Ag^−1^), coulombic efficiency after ∼1600 cycles is 94% have been also reported [29]. Furthermore, the porous nanotubes composed of spinel as an electrochemical capacitor electrode with interesting capacitance 1647 Fg^−1^ (at 1.0 Ag^−1^) and good capacity retention 77.3% (at 25.0 Ag^−1^) show 6.4% capacitance loss after 3000 cycles [30].

**Scheme 1:**
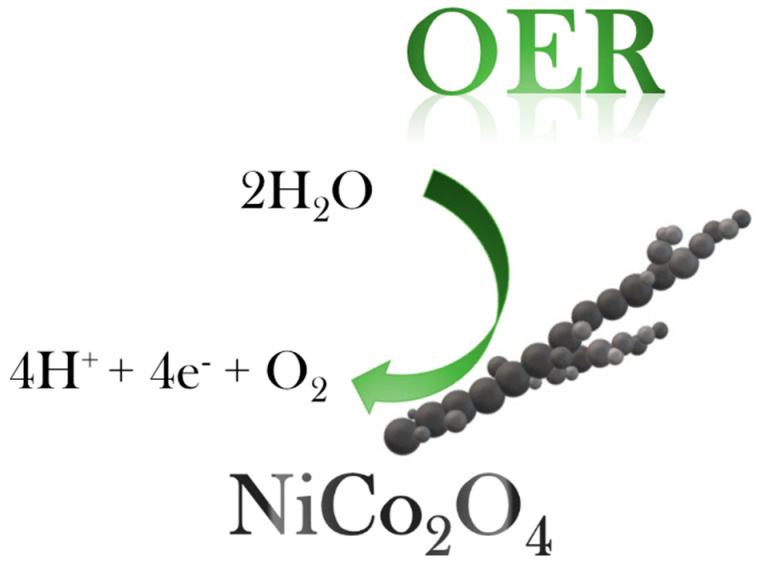
The graphical representation of the structure used in OER process.

This paper presents a simple synthesis of a NiCo_2_O_4_ spinel oxide via a one-pot solvothermal process. Nickel and cobalt precursors were picked for their high theoretical capacitance and similar potential during electrochemical reactions [31]. Our TMO particles have interesting architecture, representing spheres aggregated into rods. Keeping in mind the shape-dependent electrochemical performance of NiCo_2_O_4_ in OER, we provide a detailed characterization of rod-like materials thermally treated via pyrolysis, and carbonization, respectively. Scheme 1 presents a graphical representation of the structure used in the OER process.

## 2. Materials and Methods

All reagents were used as received (in analytical grade). Ethylene glycol (EG) purchased from Chempur (Piekary Śląskie, Poland), was used as a solvent, nickel (II) acetate tetrahydrate (Ni(OCOCH_3_)_2_·4H_2_O, NA) and cobalt (II) acetate tetrahydrate (Co(OCOCH_3_)_2_·4H_2_O) both from Sigma Aldrich (Poznań, voivodeship: Wielkopolskie, Polska, Poland) were used as a metal precursor.

### 2.1. Synthesis of NiCo_2_O_4_

Ni(OCOCH_3_)_2_·4H_2_O (257 mg) and Co(OCOCH_3_)_2_·4H_2_O (498 mg) were dissolved in the 230 mL of EG via the ultrasonication. Next, the homogeneous solution was poured into a round-bottomed flask. Then, the solvothermal reaction was carried out under reflux at 180 °C for 12 h. After that, the product was washed with pure ethanol and decanted using an ultracentrifuge. The grey, tiny rod-like precipitate, which shown magnetic responses to the magnet, was dried at 60 °C for 12 h.

The second step was the thermal decomposition. As the product, NiCo_2_O_4_ was obtained after heating in a tube furnace. The calcination process was conducted for 4 h at 300 and 400 °C in the air (100 sccm). The obtained samples were marked as NiCo_2_O_4__a300, and NiCo_2_O_4__a400, respectively.

To investigate the effect of higher temperatures on the formation of a spinel structure, a carbonization process was also carried out for 4 h at 600 and 800 °C in an inert gas (100 sccm). The samples were named NiCo_2_O_4__i600, and NiCo_2_O_4__i800.

### 2.2. Characterization

The samples’ morphology, geometry, and structure were analyzed with scanning electron microscopy (SEM, VEGA3 TESCAN, accelerating voltage at 100 kV, Tescan, Brno, Czech Republic). The characterization of chemical composition in the samples was investigated on the Raman spectroscopy (InViaRenishaw, Wotton-under-Edge, UK, laser 785 nm). The information about the crystallographic of the as-synthesized materials was provided by X-ray diffraction (XRD, AERIS PANalytical X-ray diffractometer with Cu-Kα radiation, Malvern, UK).

### 2.3. Electrochemical Measurement/Reaction Mechanism

Electrochemical tests were made in a 3-electrode glass cell, by potentiostat station (BioLogic VMP-3, Seyssinet-Pariset, France), at the constant temperature of 25 ± 0.05 °C. The system was thermostated by a temperature control bath (Hubner KISS 6, Kassel, Germany). Short-time tests were carried out with an inert gas (Ar) electrolyte purging. 

The working electrode (WE) potential was referred to the electrode made of mercury oxide (MOE) Hg|HgO, in alkaline electrolyte KOH (1 M solution). The measurements were calibrated to the Reversible Hydrogen Electrode (RHE) potential calculated by the following equation: $E_{\mathrm{RHE}}=E_{\mathrm{MOE}}+0.128+0.059\cdot \mathrm{pH}\left(V\right)$, where E_RHE_ is potential versus RHE and E_MOE_ is potential versus MOE. In order to obtain the value of the overpotential (η), the thermodynamic value of the oxygen reduction potential (i.e., 1.23 V) is subtracted from the experimental potential value. Platinum wire with the surface area of ~5 cm^2^ served as the counter electrode (CE).

The working electrode (WE) was made of 10 mm × 10 mm with 125 μm thickness graphite foil (99.8%, GoodFellow, Hamburg, Germany). The active material was dispersed under ultra-sonification in a solution made of 20% of isopropanol/water with 0.05% of Nafion. The dispersion with final concentration 10 mg/mL was applied dropwise by volume 10 μL on each side on the surface of the WE and dried for 12 h at the 60 °C.

**Scheme 2:**
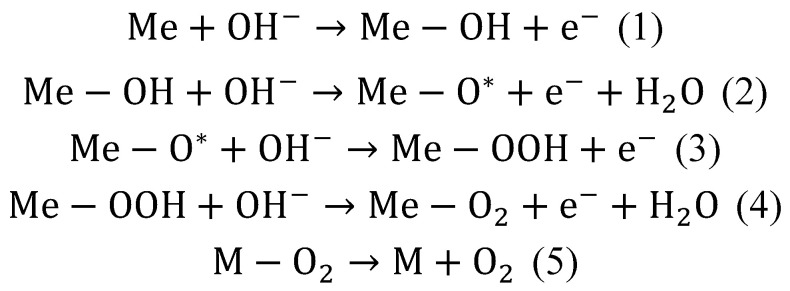
The pathways of reactions during OER of spinels particles [17].

In general, during OER, there is a loss of electrons from water molecule (H_2_O) or hydroxide ion (OH^−^) and generating oxygen (O_2_). The mechanism of this process on electrodes made of spinel particles is shown in the scheme below (see Scheme 2). The first step is the adsorption, followed by the discharge of the OH^−^ at the surface of a metal catalyst (Me) (1). The next step is the reaction of absorbed OH^−^ anions with the OH particles. The result is the production of H_2_O and adsorption of atomic O* with simultaneous electron releasing (2). The next step is the reaction between O* atom and OH^−^ (3). The result is the formation of absorbed OOH particles. It is assumed that this is the step that limits the rate of the OER. Subsequently, the reaction with additional OH^−^ leads to form an adsorbed molecules of H_2_O and O_2_ (4). The final step is the desorption of the O_2_ (5) [17]: 

## 3. Results and Discussion

**Figure 1:**
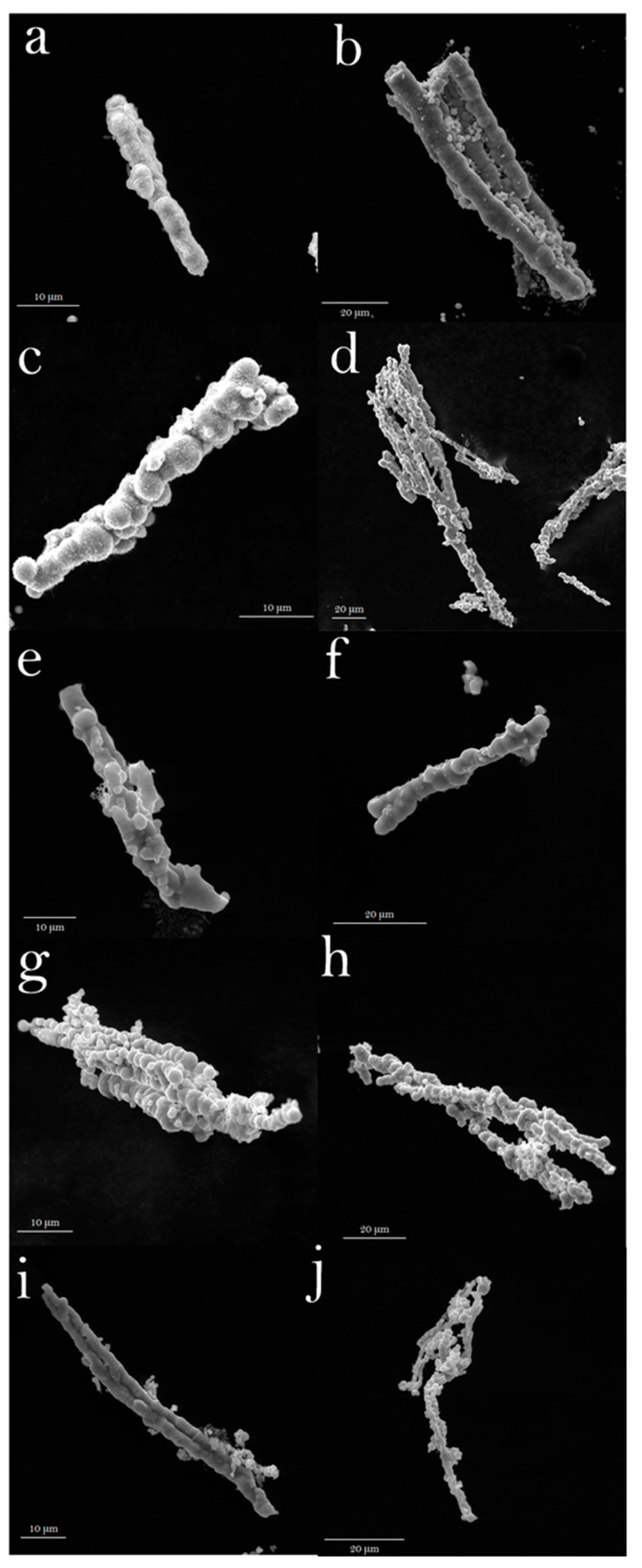
SEM images of samples: After solvothermal reaction (**a**,**b**), and after calcination: at 300 °C (**c**,**d**), and at 400 °C (**e**,**f**), after carbonization: at 600 °C (**g**,**h**) and at 800 °C (**i**,**j**).

The architecture and morphology of the samples were observed via SEM. The obtained results are presented in Figure 1 and they clearly show that all the samples exhibit the rod-like structure which is made of tiny spheres with diameters of ~1.7 µm, ~4.3 µm, ~2.6 µm, ~3.0 µm, and ~1.8 µm, for precursor, pyrolyzed at 300 °C, 400 °C, carbonized at 600 °C, and 800 °C, respectively. The length of rods are ~52.2 µm, ~42.6 µm, ~ 50.9µm, ~90.0 µm, and ~51.3 µm, for precursor, pyrolyzed at 300 °C, 400 °C, carbonized at 600 °C, and 800 °C, respectively. The diameters of rods are estimated to be ~4.1 µm, ~5.8 µm, ~5.7 µm, ~4.5 µm, and ~1.9 µm, for precursor, pyrolyzed at 300 °C, 400 °C, carbonized at 600 °C, and 800 °C, respectively. Interestingly, the material retained the original shape of the particles after thermal treatment. The shape of the obtained spinels may result from the type of synthesis method used or the presence of additional templates. Devaguptapu et al. obtained typical NiCo_2_O_4_ spinel needle-like shapes and then modified them using various templates [32]. These needles consist of many nanoparticles with a size around 10–20 nm and have a greater tendency to aggregate in comparison to our material. Authors also proved that the electrochemical performance is high morphology-dependent. NiCo_2_O_4_ spinel can also take the form of nanowires. Jin et al. [33] synthesized tangled together nanowires composed of many nanoparticles. A similar morphology was repeated several more times. The urchin-like spheres composed by NiCo_2_O_4_ were obtained by Qin et al. [34]. In this case, samples have inverse geometry, where the spheres are made up of nanowires. Resembling morphology was reported by Chen et al. [35]. They obtained rambutan-like spinels microspheres. The same architecture has been reported by Li et al. [36]. The product has also shown the morphology of nanoneedles. This architecture was assembled radiantly into a rambutan-like structure. In our case, the structures are isolated, but are not as thin as wires and needles, and the rods are more like branches made of many spheres.

**Figure 2:**
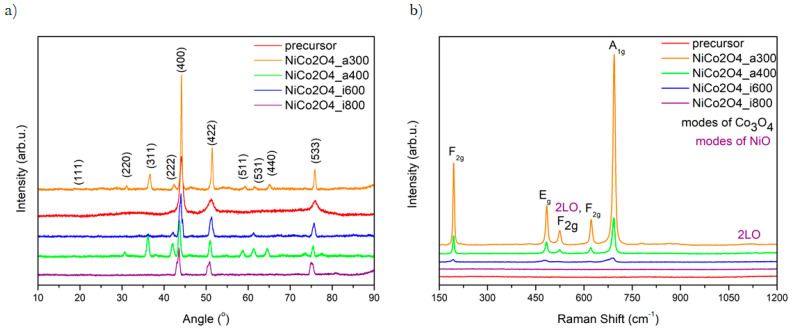
XRD patterns (**a**) and Raman Spectra (**b**) of obtained samples.

The crystal structure of the metal oxide was analyzed using X-Ray Diffractometry (XRD) (Figure 2a). All reflections are well-indexed to NiCo_2_O_4_ cubic structure, confirmed by referencing with JCPDS card No. 20-0781. All diffractograms contain reflections at 2θ values of 20.14°, 30.59°, 36.11°, 41.96°, 43.54°, 50.82°, 58.72°, 61.42° and 75.48°. These values of degrees are corresponding to (111), (220), (311), (222), (400), (422), (511), (531), (440), and (533), respectively [37,38]. Furthermore, the XRD pattern shows reflections attributed to other species: Co_3_O_4_, and NiO. It has been referred with JCPDS card no. 43-1003, and 47-1049, respectively. These results clearly demonstrate Ni and Co’s influence on the spinel structure and have been reported before [39,40]. The purity of samples was proved by the absence of any unidentified peak. The results confirm that 300 °C is high enough to obtain the crystalline NiCo_2_O_4_ spinel. Moreover, the calcinated samples have shown higher peaks than carbonized ones. This is due to the existence of a more significant number of oxygen vacancies. An increasing number of these vacancies is caused by the different partial pressure of the oxygen particles, which leads to a pronounced effect on the oxygen evolution [41].

The results obtained via Raman Spectroscopy are shown in Figure 2b. They present the vibronic properties and composition changes in the structure of the synthesized samples. The main peaks corresponding to the modes of spinel structure are in the range between 190 and 800 cm^−1^ and they are detected in each sample. A mode can be defined as an excitation state of a standing wave in a dynamical system. Specific one or few frequencies characterize each of them, so each store has a defined amount of energy. The modes are labelled by different letters (e.g., A, B, E, L), which assign vibrational movement to a particular group of points [42]. The highest intensity of peak is presented in samples calcinated at 300 °C. The height of the local maximum decreases with increasing temperature. The spectra show vibrational peaks precisely at 196, 482, 522, 621, and 692 cm^−1^, which correspond to F_2g_, E_g_, F_2g_, F_2g_, and A_1g_ Co_3_O_4_ modes, respectively [43]. The A_1g_ mode is assigned to the vibrational moves of ions placed in octahedral sites, and the peaks at E_g_ and 522 cm^−1^ from F_2g_ mode are due to the occurring simultaneously vibrations of both octahedral and tetrahedral oxygen atoms in the crystal structure. Two Raman peaks present in the spectra at ~522 and 1100 cm^−1^ are assigned to NiO shaking peaks. The appearance of local maximum could be defined as the longitudinal optical (1LO) phonon modes of, and two-phonon (2P) 2LO modes of this compound [44]. The highest peaks in the pyrolyzed samples suggest that the air is the best thermal decomposition environment forming a well-crystallized structure: The higher peak, the stronger interaction between atoms Ni-Co-O [45]. Additionally, the Raman spectrum of the sample pyrolyzed at 300 °C exhibits only Co–O and Ni–O vibrational maximum, which proves the successful decomposition of both nickel and cobalt precursors, leading to form NiCo_2_O_4_. XRD data are in full agreement with Raman investigations.

OER is a complementary reaction for HER, which proceeds simultaneously on CE. The prepared electrodes’ electrocatalytic properties were tested in OER process, due to its higher energy barrier, which makes it rate-limiting process during water splitting. ORR is the reverse electrochemical reaction that takes place in fuel cells. The measurements were collected and performed by Linear Sweep Voltammetry (LSV). The main element of the system is a 3-electrode cell. As an electrolyte was used KOH (1 M concentration The sweep speed was at the level 5 mVs^−1^, an IR drop factor made corrections. The surveys were carried out in order to achieve the absolute value of current density -25 mAcm^−2^ and potential below the standard potential during OER related to RHE (1.229 V vs. RHE). The data were collected to evaluate the samples’ activity as the electrocatalysts, via its value of overpotential η [mV]. That value is a difference between thermodynamics in HER/OER at a specific current density. Mostly it is measured at 1 and 10 mAcm^−2^, which is identified as η_1_ and η_10_, respectively. A better reaction rate is indicated by lower η value, which means the best catalytical activity has the lowest value of η.

**Figure 3:**
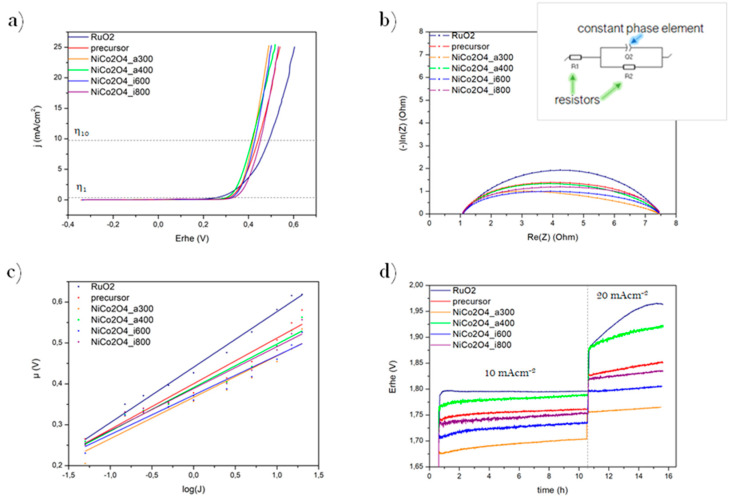
OER electrochemical measurements: LSV plots (**a**), Nyquist plots (with an inset graph window of equivalent Circuit R1R2Q) (**b**), Tafel plots (**c**), and chronopotentiometry plots (**d**) of the obtained materials compared to RuO_2_.

The performances of the overpotential during LSV test in the OER process are included in and Figure 3a and Table 1. That the onset overpotentials of synthesized samples at a current density of 1 and 10 mAcm^−2^ are lower than or comparable to RuO_2_, indicating great electrochemical activity. At a current density of 1 mAcm^−2^ the best performance exhibits the NiCo_2_O_4__a400 (with η_1_ 326 mV). The NiCo_2_O_4__a300 and NiCo_2_O_4__a400 showed the best electrocatalytic performance at a current density of 10 mAcm^−2^ (both η_10_ 420 mV). However, it is essential to point out better results of all spinel samples than RuO_2_, implying the critical role of high catalyst conductivity in the OER process.

The next measurement was carried out to collect accurate data about the electrochemical activity of catalysts via Galvanostatic Electrochemical Impedance Spectroscopy (GEIS). It was applied at current density 5 mAcm^−2^ with an amplitude of 10 mV at the frequency range between 100 mHz and 100 kHz. They were fitted on the BioLogic software to the equivalent circuit (R_1_ + R_2_)/Q via Z-fit electrochemical fitting program (Figure 3b). That circuit was chosen because it is the simplest one suitable for the measurement system. It contains, R1 which is the electrolyte resistance [Ω], which is linked in series with R_2_—resistance of transfer an electric charge [Ω]. It is also connected in parallel with the element of constant phase Q (Fs^(a−1)^)^−1^. Real resistance R [Ω] is the abscissa value of the starting point at the diagram and it starts at the maximum of frequency. It represents the sum of the resistance of all connectors and electrolyte. All these elements are linked together in series (marked as a R_1_ resistor on the scheme). The diameter of the half-circle is calculated from points at X-axis. This parameter is the representation of the charge transfer resistance value (marked as R_2_). Its vertical size is indicated as a Q element, representing a constant phase. The more developed the catalyst surface, the greater value of Q. The difference between diameters of semi-circles are results of a change in R_2_ value, which is proportional to the inverse value of the intensity of the HER/OER process taking place on the electrode. In other words, lower R_2_ value indicates the better activity of the electrocatalyst (under the same process conditions).

The differences in equivalent resistance during the OER process are implied in Figure 3b and Table 1. Presented Semi-circles have a similar shape and start in the same value in the abscissas axis. All samples have lower R_2_ than RuO_2_, which confirms the high activity of the synthesized materials. The lowest result indicates the best activity of 3.52 Ω is exhibited by NiCo_2_O_4__a300, which suggests a better electrical conductivity and faster charge transport of the electrode.

For a better description of the electrochemical evolution process Tafel equation via steady-state chronopotentiometry (CP) have been revealed. For calculations was used a simple dependency: f(x) = η = f(log(j)). The measurement of potentials on the WE occurs while maintaining different direct current (DC) for a certain time. Then the value of the Tafel slope is calculated using the linear regression method. The lower value of slope means a better catalyst activity, due to a faster electrochemical reaction rate.

The results of Tafel properties toward the OER process are shown and summarized in Figure 3c and Table 1. All the samples have lower slope values than RuO_2_, indicating more effective electron transfer, and better catalytic activity during OER for NiCo_2_O_4_ spinel. The electrode composed of NiCo_2_O_4__a300 has the most favorable electrocatalytic reaction kinetics, represented by the lowest value of the slope equal to 3.52 mVdec^−1^.

Finally, for a complete description of the electrocatalytic activity, the stability tests have been conducted. The material used as electrocatalyst should exhibit high electroactivity and great durability in a long operation, which means that the minimum difference in overpotential is desirable. Chronopotentiometry method was used to examine the performance of samples. The measurement was performed in two stages: first stability was tested at a current density of 10 mAcm^−2^ for 10 h, followed by the second step at 20 mAcm^−2^ for the next 5 h. Relative changes in overpotentials over time was calculated with the following formula:

(1) $$\left|\frac{f\left(x_{1}-x_{0}\;\right)}{f\left(x_{0}\right)}\times 100\%\right|=\Delta \eta$$

where x_0_ is the starting point of the plateau, and x_1_ is the finishing point.

During the first step of a stability test (Figure 3d and Table 1), the overpotential exhibited by RuO_2_ has the lowest relative growth (+0.56%), and among the obtained samples, the most stable turned out to be NiCo_2_O_4__i800 (+0.57%). During the second step—at higher current density, the best stability performance, with constant overpotential, has been exhibited by NiCo_2_O_4__i600. The stability chronopotentiometry long tests show that NiCo_2_O_4_ spinel particles are excellent candidates to use as OER catalyst material.

The present state of the art report that the electrochemical performance of NiCo_2_O_4_ spinels in OER is shape-dependent. Table 2 presents the collected data on the main OER results measured with NiCo_2_O_4_ spinels with different architecture such as nanoflowers, nanospheres, nanoparticles, nanoflakes, and nanoflowers [46,47,48,49,50,51,52]. The main parameters are η at 10 mAcm^−2^, Tafel slope and stability tests. However, not many contributions report the results on stability tests. Comparing them to our data shows that the proposed samples of spinels, with a rod-like structure composed by aggregated spheres, behave in a stable manner, even in the long-term. It can be further concluded that the synergy of spheres and rods provided efficient electrocatalysts with superior stability and promising activity. As mentioned before, Hf nanoparticles have been recently reported as a novel electrocatalyst with relatively low values of overpotential at 10 mAcm^−2^ (358 mV) and Tafel slope of 85 mV/dec [5]. Therefore, it is desired to study these materials in greater detail and various morphology to reveal a clear correlation between spinels shape and their electrochemical performance. 

## 4. Conclusions

In conclusion, NiCo_2_O_4_ rod-like catalysts were successfully fabricated in a two-step process: Solvothermal reaction and annealing route as a facile route to synthesize spinel particles. The results collected from X-ray diffractogram and Raman spectroscope affirmed the formation of nickel-cobalt oxide compounds. These samples, with novel morphology, exhibited outstanding catalytic activity in the oxygen evolution reactions. Fabricated spinel material showed excellent electrochemical properties for the OER, better than commercial RuO_2_: It is due to the presence of a high quantity of metals nanoparticles. A different condition during thermal treatment has been investigated to compare the influence of temperature, as well as the gas atmosphere on electrocatalytic activity. All samples were tested in a variety of electrochemical measurements during the OER process. As all summarized results have shown, the best electrochemical performance is exhibited by NiCo_2_O_4__a300 sample, which has high activity (due to low overpotential η_1_ = 346, η_10_ = 420 mV, low Tafel slope = 101 mV/dec, and equivalent resistance R_2_ = 3.52 Ω) as catalysts and almost constant overpotential during a long-term test (high persistence at level ~98.81% during I step, and ~99.43% during II step), which demonstrate the crucial roles of oxygen vacancies, which are created with larger amount during annealing under air atmosphere.

## Figures and Tables

**Table 1:** Electrochemical properties of the obtained samples and RuO_2_ during OER process: overpotential η_1_ and η_10_, Tafel slope, R_2_ equivalent resistance values, and relative changes in overpotentials over time ∆η.

Sample		η_1_ [mV]	η_10_ [mV]	Tafel Slope [mV/dec]	R_2_ [Ω]	∆η_10_ [%]	∆η_20_ [%]
RuO_2_		312	491	136	6.40	0.56	4.26
Precursor		340	446	112	5.35	1.15	1.09
NiCo_2_O_4_	a300	346	420	101	3.52	1.19	0.57
	a400	328	420	106	4.85	1.13	2.13
	i600	346	430	97	3.89	1.75	0.00
	i800	359	454	103	4.95	0.57	0.55

**Table 2:** Comparison of morphologies and catalysts activity exhibited by NiCo_2_O_4_ spinel.

Architecture of the Sample	η [mV] (at 10 mAcm^−2^)	Tafel Slope [mV/dec]	Persistence of Overpotential [%]	References
Rod-like	420	97	99 (10.5 h, 10 mAcm^−2^) 100 (5 h, 20 mAcm^−2^)	This work
Nanoflowers	383	137	47 (1 h, 6.8 mVcm^−2^)	[46]
Hollow Nanospheres	428	141	31 (1 h, 3.5 mVcm^−2^)	
	520	150	96 (12 h, 1.76 V)	[47]
Urchin Nanospheres	441 (at 5 mAcm^−2^)	103	91 (12 h, 1.76 V)	
Nanoparticles	422	136	-	[48]
Nanoflakes	500	119	-	[49]
	430	76	87 (24 h, 10 mAcm^−2^)	[50]
	360	131	-	[51]
Nanowires	271	172	-	[52]
